# High-grade prostatic stromal sarcoma with a distinctive cystic-solid MRI pattern and aggressive clinical course: a case report

**DOI:** 10.3389/fonc.2026.1762973

**Published:** 2026-03-03

**Authors:** Xiaoyan Lei, Kehui Liu, Fujin Liu, Shishi Luo

**Affiliations:** 1 Department of Radiology, Hainan General Hospital (Hainan Affiliated Hospital of Hainan Medical University), Haikou, Hainan, China; 2 Department of Radiology, Haikou Hospital of Xiangya Medical College, Central South University, Haikou, Hainan, China; 3 Department of Pathology, Hainan General Hospital (Hainan Affiliated Hospital of Hainan Medical University), Haikou, Hainan, China

**Keywords:** case report, high-grade, MRI, prostate, PSS, stromal sarcoma

## Abstract

**Objectives:**

To report a rare case of high-grade prostatic stromal sarcoma (PSS) and delineate its characteristic magnetic resonance imaging (MRI) features to facilitate early detection and accurate diagnosis.

**Methods:**

A 55-year-old male presented with progressive dysuria. Preoperative multiparametric pelvic MRI was performed. The imaging findings were correlated with histopathology from a robot-assisted laparoscopic radical prostatectomy.

**Results:**

MRI revealed a large, well-demarcated, encapsulated cystic-solid mass within the prostate. The solid components showed restricted diffusion. The cystic component contained multiple septations and demonstrated no diffusion restriction. An involvement nodule was identified in the right seminal vesicle. Histopathology confirmed high-grade prostatic stromal sarcoma with positive surgical margins. The patient experienced local recurrence within six months despite adjuvant chemotherapy.

**Conclusions:**

Prostatic stromal sarcoma is a rare malignancy with distinct MRI features that can help differentiate it from more common prostatic neoplasms.

## Introduction

Prostatic stromal sarcoma (PSS) is a malignant tumour originating from the specialised prostatic stroma, first systematically classified by Gaudin et al. in 1998 into stromal tumours of uncertain malignant potential (STUMP) and prostatic stromal sarcomas (PSS) (1). PSS is exceptionally rare, constituting only about 0.1% of all primary malignant prostate tumours in adults (2). Its clinical presentation is non-specific, often leading to misdiagnosis as benign prostatic hyperplasia or more common malignancies. Preoperative diagnosis is challenging, and prognosis is generally poor, especially for high-grade variants. This paper presents a case of high-grade PSS, with a particular focus on its distinctive MRI characteristics, aggressive clinical course, and the critical lessons it offers for diagnosis and management, contextualised within the existing literature.

## Case report

### Clinical data

A 55-year-old male presented with a one-month history of progressive dysuria, characterised by poor urine flow, hesitation, and a sensation of incomplete emptying. Digital rectal examination revealed a symmetrically enlarged prostate (consistent with Grade II enlargement), which was diffusely firm in consistency. Its surface was smooth without discrete nodules or areas of induration. The overlying rectal mucosa was smooth and freely mobile. Laboratory investigations, including complete blood count and renal function tests, were within normal limits. Notably, both total and free prostate-specific antigen (PSA) levels were normal (tPSA 0.743 ng/mL, fPSA 0.211 ng/mL). The patient had no significant prior medical or surgical history related to the genitourinary system, and no relevant family history was reported.

### Timeline

The key events in this case are summarized as follows:

Month 0: Onset of progressive dysuria.

Month 1: Clinical presentation, normal PSA, transabdominal ultrasound, and multiparametric MRI performed. Findings suggestive of malignant prostatic mass with seminal vesicle involvement.

Month 1 (shortly after diagnosis): Robot-assisted laparoscopic radical prostatectomy performed.

~40 days post-surgery: Commencement of adjuvant chemotherapy (pirarubicin and ifosfamide). The patient completed the planned initial cycles without reports of major acute toxicity requiring hospitalization.

6 months post-surgery: Follow-up CT scan revealed local recurrence at the surgical bed.

### Imaging findings

**Figure 1:**
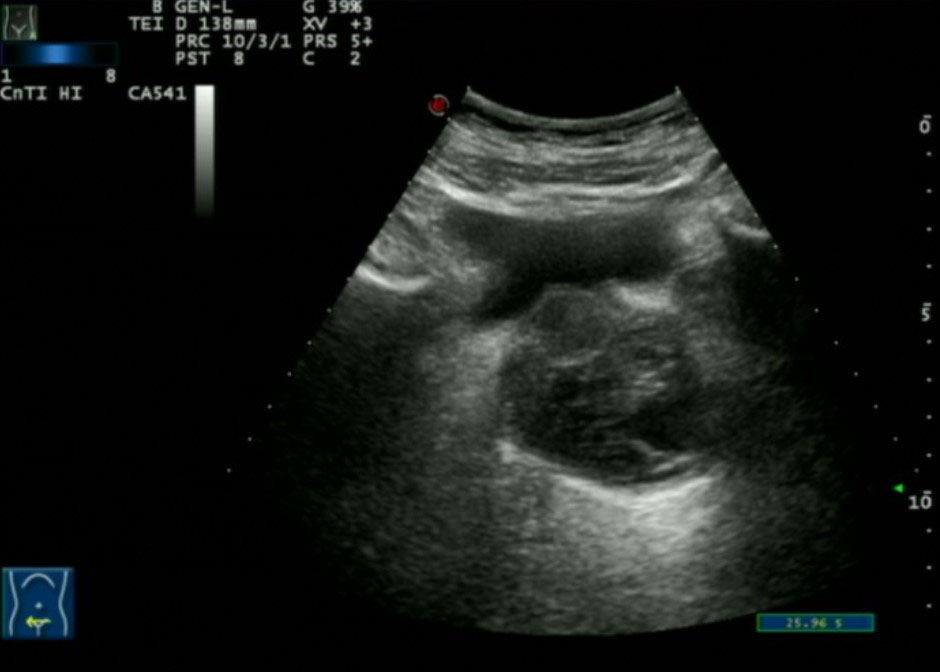
Prostate ultrasound showing an enlarged prostate with an intact capsule. The parenchyma exhibits heterogeneous echogenicity with patchy areas of strong echoes. A cystic-solid mixed echogenic mass, measuring approximately 5.0 x 4.8 cm, with unclear boundaries and heterogeneous internal echoes, is observed within the prostate.

Transabdominal ultrasound showed an enlarged prostate containing a mixed echogenic mass with poorly defined boundaries and no significant vascularity on Doppler imaging (**Figure 1**).

**Figure 2:**
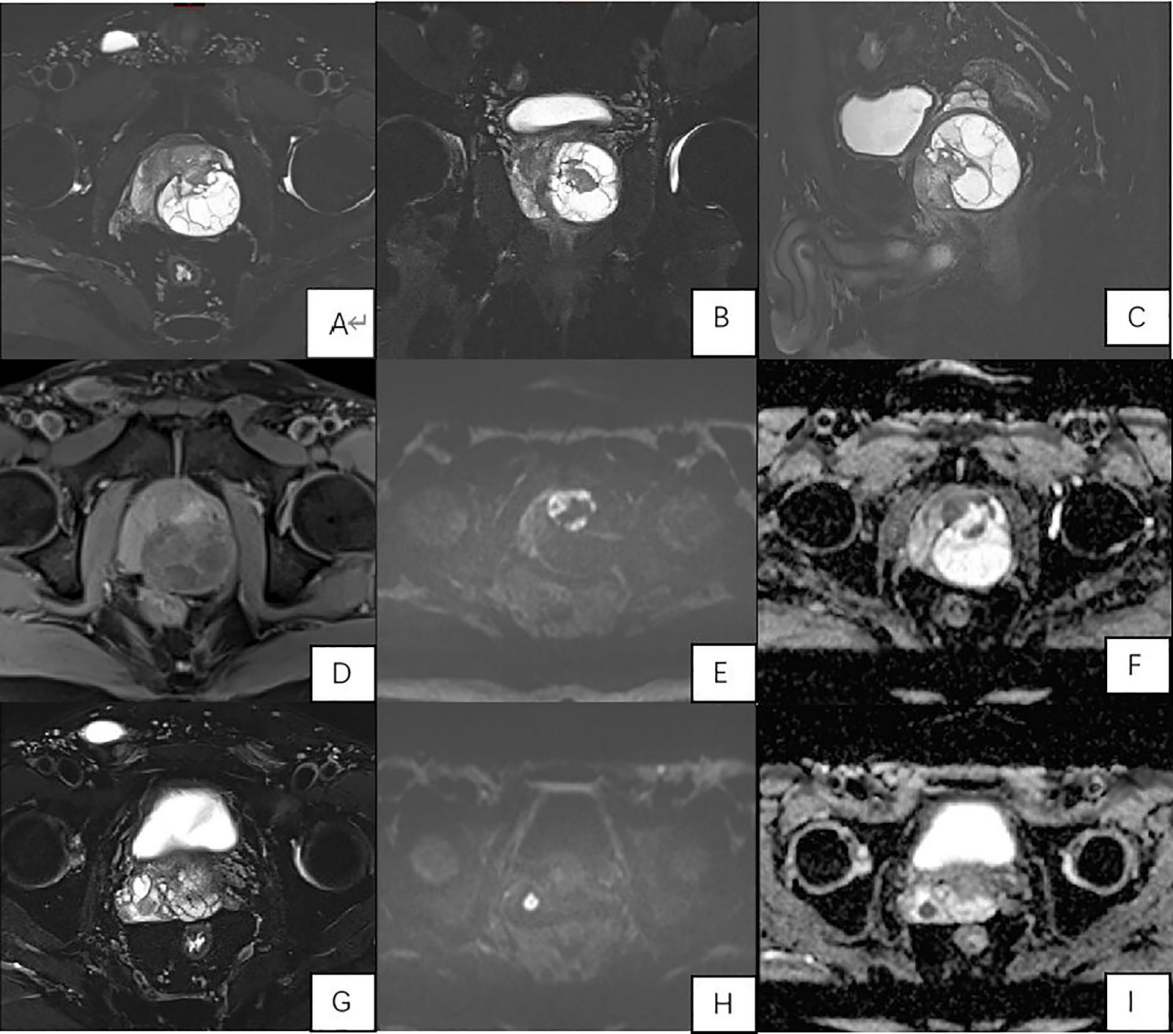
Multiparametric MRI of the Prostate. **(A-C)** (T2-weighted images): Axial **(A)**, coronal **(B)**, and sagittal **(C)** views show a large, encapsulated cystic-solid mass. The cystic component is T2-hyperintense with internal septations. The solid component is T2-hypointense; **(D)** (T1-weighted image): The solid component is iso-intense; **(E)** (DWI, b=2000): The solid component and the seminal vesicle nodule show high signal, indicating restricted diffusion; **(F)** (ADC map): Corresponding low signal in the solid component and seminal vesicle nodule confirms restriction; **(G-I)** (Seminal Vesicle Metastasis): T2-weighted image **(G)**, DWI **(H)**, and ADC map **(I)** clearly depict the involvement nodule in the right seminal vesicle.

Multiparametric pelvic MRI (3T Siemens Prisma) was subsequently performed. It revealed a large prostate (6.0 cm x 5.2 cm x 5.7 cm) harbouring a well-demarcated, encapsulated cystic-solid mass measuring approximately 4.9 cm x 4.4 cm in the left lobe. The solid anterior component showed iso-intense T1 and slightly hypointense T2 signal with restricted diffusion (high signal on DWI (b=2000 s/mm²) and low signal on the ADC map). The larger cystic component extended posteriorly, demonstrating T2 hyperintensity, multiple internal septations, and no diffusion restriction. A critical finding was a nodular lesion in the right seminal vesicle with imaging characteristics identical to the solid tumour component, highly suggestive of metastasis (**Figure 2**).

### Diagnostic pathway and decision for surgery

Given the patient’s normal serum PSA level, the presence of a large, complex cystic-solid prostatic mass on MRI was highly atypical for common prostatic adenocarcinoma. The imaging features—particularly the well-defined capsule, internal septations, restricted diffusion in solid components, and apparent involvement of the right seminal vesicle—raised a strong suspicion of an aggressive mesenchymal neoplasm, such as a prostatic stromal sarcoma or leiomyosarcoma. A preoperative biopsy was considered. However, after multidisciplinary discussion, it was decided to proceed directly to radical prostatectomy for the following reasons: (1) the MRI findings were highly suggestive of a malignant, locally advanced tumour; (2) given the cystic nature of the mass, there was a concern about potential seeding or inadequate sampling via biopsy; and (3) the patient’s symptoms were progressively worsening, warranting definitive surgical intervention for both diagnosis and potential cure.

### Surgical and histopathological findings

**Figure 3:**
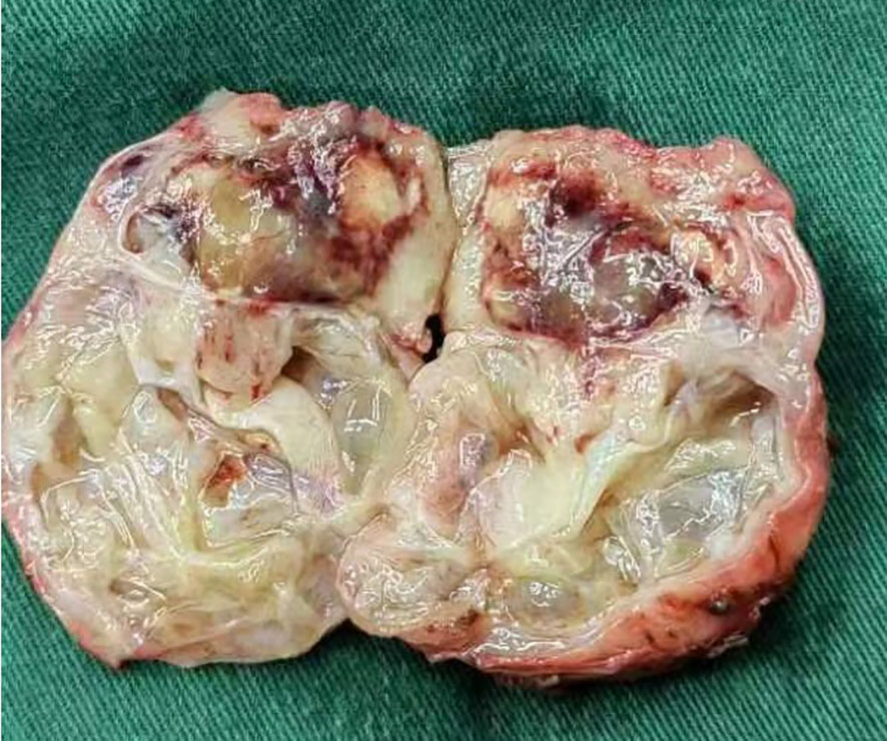
Gross pathology specimen of the resected prostate shows a well-circumscribed, nodular mass with a variegated cut surface. Cystic areas are evident.

Given the MRI findings, a robot-assisted laparoscopic radical prostatectomy was performed. Intraoperatively, a cystic-solid mass adherent to the prostate surface was noted. Gross pathological examination revealed a 5.1 x 4.6 x 4.5 cm nodular mass with a grey-yellow cut surface and multilocular cystic areas containing clear fluid (**Figure 3**).

**Figure 4:**
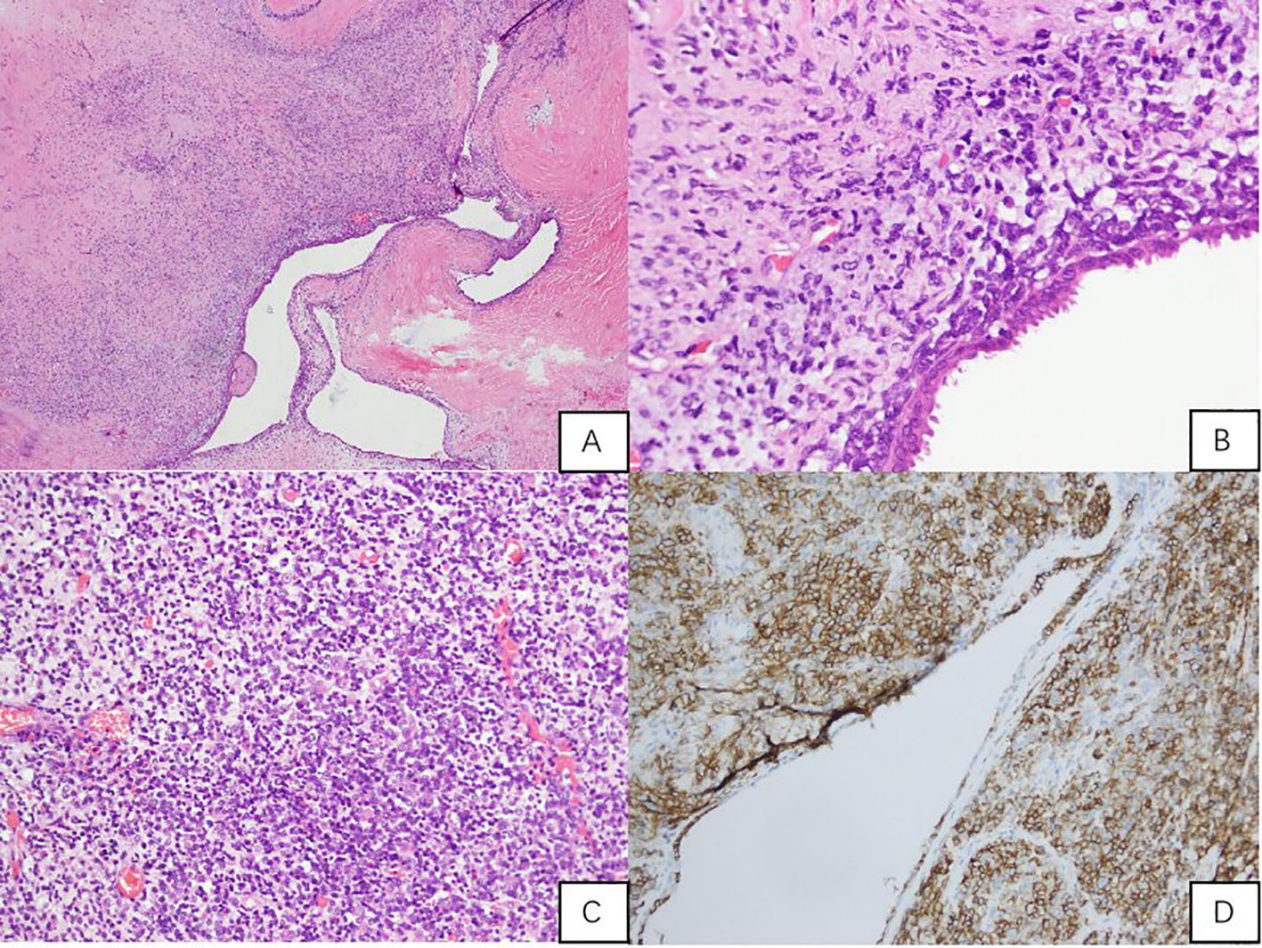
Histopathological and Immunohistochemical Analysis. **(A)** (H&E, x40): Sarcomatoid stroma with invaginated benign prostatic glands, forming a complex, leaf-like architectural pattern (phyllodes-like pattern); **(B)** (H&E, x200): Under the epithelium, atypical short spindle-shaped and oval tumour cells proliferate, with frequent mitoses and stromal hyaline degeneration; **(C)** (H&E, x200): Stroma-rich area with deeply stained, small nuclei tumour cells, showing frequent mitoses; **(D)** (IHC for CD56, x200): Stromal tumour cells CD56 positive.

Microscopically, the tumour was composed of densely packed, atypical spindle cells arranged in a phyllodes-like pattern around benign prostatic glands. There was significant nuclear pleomorphism, frequent mitotic figures, and foci of necrosis. Immunohistochemistry was positive for Vimentin and CD56, The tumour was negative for epithelial markers (PSA, P504S, CK, CK34βe12), neuroendocrine markers (Syn, CgA, NSE, INSM1), melanocytic markers (S-100, HMB45, MelanA), haematolymphoid marker (LCA), androgen receptor (AR), and α-inhibin. β-Catenin showed cytoplasmic positivity. The Ki-67 proliferation index was high (approximately 50% in hotspots). CD117 and DOG1 were not performed due to the morphological and immunophenotypical features being inconsistent with gastrointestinal stromal tumour (GIST), and the limited availability of these specific markers in our institution’s routine diagnostic panel at the time of assessment. Nevertheless, the overall immunoprofile supports the diagnosis of high-grade prostatic stromal sarcoma. The final diagnosis was high-grade prostatic stromal sarcoma with positive surgical margins (**Figure 4**).

### Postoperative course and follow-up

The patient’s initial recovery was unremarkable. However, forty days post-surgery, he commenced adjuvant chemotherapy (pirarubicin and ifosfamide). He tolerated the treatment regimen without documented severe adverse events during the initial cycles. Despite adjuvant chemotherapy, a follow-up CT scan at six months revealed local recurrence at the surgical bed with increased involvement of the right seminal vesicle and adjacent musculature. This rapid progression represented a significant adverse outcome.

### Patient perspective

The patient expressed profound distress upon learning the diagnosis of a rare and aggressive cancer. He reported anxiety regarding the prognosis and the potential impact on his quality of life. He was committed to the proposed treatment plan, hoping for curative intent. Following surgery and during chemotherapy, he experienced expected fatigue but was grateful for the coordinated care from the urology and oncology teams. The news of early recurrence was met with disappointment, and he agreed to continue with further multidisciplinary management discussions.

## Discussion

Prostatic stromal sarcoma remains a diagnostic and therapeutic challenge in clinical practice. Our case of high-grade PSS exemplifies its aggressive behaviour and provides an opportunity to refine its imaging diagnosis and management principles. While previous reports have established the fundamental clinicopathological profile of PSS (1–3), our case elucidates several unique and underemphasised features that merit detailed discussion.

### Distinctive imaging pattern and diagnostic implications

The MRI presentation in our case—a large, well-demarcated, encapsulated cystic-solid mass with internal septations and restricted diffusion in the solid components—offers a valuable template for identifying PSS. While previous cases have described large prostatic masses with cystic change (4–11), the presence of a defined capsule and multiple internal septations is a noteworthy detail that is underemphasised in the literature. This appearance can be misconstrued as a benign entity, such as a complex cyst or cystadenoma. However, the combination of these features with restricted diffusion in the solid nodules and evidence of extraprostatic extension (in our case, to the seminal vesicle) creates a highly suspicious profile for a malignant stromal tumour.

This constellation of findings is distinctly different from the more common prostate adenocarcinoma, which typically lacks prominent cystic components and a well-defined capsule, and usually originates in the peripheral zone. Our case, therefore, underscores that when a complex cystic-solid mass with an apparent capsule and septations is encountered in the prostate of a middle-aged man with normal PSA, PSS should ascend in the differential diagnosis, ahead of more common entities.

### Aggressive biology and management challenges

The clinical course of our patient starkly demonstrates the aggressive biology of high-grade PSS. Despite a macroscopically complete robotic resection, and adjuvant chemotherapy, local recurrence at the surgical bed (with positive margins) was detected within six months, aligning with the known poor prognosis of high-grade PSS (10, 12, 13). This highlights a critical clinical point: even seemingly organ-confined disease on preoperative imaging can harbour microscopic spread.

Our patient’s rapid recurrence, despite adjuvant chemotherapy, invites discussion on the optimal management strategy. The choice of adjuvant chemotherapy with pirarubicin and ifosfamide was based on the regimen’s activity against high-grade soft tissue sarcomas, particularly given the tumour’s high proliferative index (Ki-67 ~50%) and aggressive histology. While no standardized protocol exists for PSS, this combination is commonly employed for other high-grade pelvic sarcomas. The literature lacks consensus, but some reports, such as that by Hodotsuka et al. (12, 14), suggest a potential benefit from neoadjuvant chemotherapy to downstage the tumour before surgery. Given the high Ki-67 index (approximately 50%) in our case, indicating high proliferative activity, a more aggressive upfront approach incorporating neoadjuvant therapy might be considered for future similar cases to improve the chances of negative margins and reduce recurrence risk. Radiotherapy may also have a role in local control, particularly in cases with positive margins (14).

### The evolving role of PSMA PET/CT in staging aggressive prostate malignancies

The staging of aggressive prostatic tumours is increasingly incorporating molecular imaging. Prostate-specific membrane antigen positron emission tomography/computed tomography (PSMA PET/CT) has become a cornerstone for staging conventional prostate adenocarcinoma due to its high sensitivity for detecting nodal and distant metastases, directly influencing risk stratification and management planning (15). However, its utility in rare, high-grade non-epithelial or variant histologies, such as prostatic stromal sarcoma, is less established and warrants caution. Importantly, PSMA expression can be heterogeneous or absent in certain high-grade phenotypes, including those with ductal differentiation or sarcomatoid features, potentially leading to false-negative findings (16). Therefore, while PSMA PET/CT should be considered in the initial staging workup of any aggressive prostate mass to evaluate for occult metastatic disease, a negative scan must not be over-interpreted as excluding clinically significant or locally advanced malignancy. In cases with highly suspicious conventional imaging (as in our patient), a multidisciplinary approach remains essential, and treatment decisions should not be deferred solely on the basis of a negative PSMA PET/CT. In our case, PSMA PET/CT was not performed due to the predominant cystic morphology and the urgent need for surgical intervention based on the compelling MRI findings and clinical progression.

### Key learning points and literature integration

This case reinforces several crucial lessons from the literature while adding new nuances: (1).Normal PSA is the Rule: As consistently reported (7) and confirmed here, a normal PSA in the setting of a large prostate mass should immediately raise red flags for non-epithelial tumours like PSS. (2).MRI is Pivotal for Characterisation: Our case powerfully illustrates that MRI is the cornerstone for diagnosis. The ability to characterise the internal architecture (cystic *vs*. solid, septations), assess diffusion characteristics, and evaluate for extraprostatic extension is unparalleled, as emphasized by Tamada et al. (17) and now further validated by our findings. (3).The “Cystic-Solid Mass with Septations” Pattern: We propose that this specific MRI pattern—a large, encapsulated cystic-solid mass with internal septations and restricted diffusion in solid components—is highly suggestive of high-grade PSS, especially when coupled with seminal vesicle invasion and normal PSA. This pattern warrants recognition, though its diagnostic specificity requires validation in larger, multi-centre cohorts. This refines the previously described imaging features of merely “large masses with necrosis”. (4). Guarded Prognosis and Need for Multimodality Therapy: The dismal outcome in our patient and others (10, 13) confirms the virulent nature of high-grade PSS. It underscores that surgery alone is often insufficient. A multidisciplinary approach involving oncology and radiation oncology from the outset, potentially employing neoadjuvant or more intensive adjuvant protocols, should be the standard of care.

### Molecular profiling and future directions

Beyond histomorphology and immunophenotype, the molecular landscape of rare prostatic malignancies like PSS remains largely unexplored. Molecular profiling, including next-generation sequencing for genetic mutations, and the evaluation of emerging biomarkers (e.g., microRNAs, gene expression signatures), holds promise for refining tumour classification, identifying therapeutic targets, and improving risk stratification (18). Although comprehensive molecular analysis was not performed in the present case due to institutional constraints—a noted limitation—future studies integrating such data are crucial.

### Limitations

This report has several limitations. First, it is a single-case report, which inherently limits the generalizability of the findings. The management strategy and outcomes are specific to this patient and institution. Second, the follow-up period is relatively short; longer-term survival data are not yet available. Third, comprehensive molecular profiling (e.g., next-generation sequencing, biomarker analysis such as microRNA signatures) was not performed on this tumour, which limits our understanding of its specific genetic drivers and potential alignment with emerging targeted therapy paradigms. This underscores the need for future studies to incorporate such analyses. Finally, while we propose a characteristic MRI pattern, its diagnostic specificity needs validation in larger, multi-centre studies.

## Conclusion

Prostatic stromal sarcoma should be a primary consideration in young to middle-aged patients presenting with a large, complex cystic-solid prostatic mass exhibiting a capsule, internal septations, and restricted diffusion, particularly in the context of normal serum PSA levels. MRI is the optimal imaging modality for suggesting this rare diagnosis. The aggressive nature of high-grade PSS, as evidenced by rapid recurrence in our case, mandates a low threshold for extensive resection and a strong consideration for a multimodality treatment strategy at the time of initial diagnosis to improve patient outcomes.

## Data Availability

The original contributions presented in the study are included in the article/supplementary material. Further inquiries can be directed to the corresponding author.
